# Determinants of crypto art adoption among Chinese consumers: an extended UTAUT2 approach

**DOI:** 10.3389/fpsyg.2026.1948079

**Published:** 2026-09-24

**Authors:** Yanying Chen, Kebing Lin, Yuanyuan Hao, Ziqin Chen

**Affiliations:** School of Architecture and Art, Central South University, Changsha, China

**Keywords:** Chinese market, consumer adoption, crypto art, digital art consumption, NFT, social influence, structural equation modeling, UTAUT2

## Abstract

**Introduction:**

Crypto art, enabled by blockchain-based non-fungible tokens (NFTs), represents a transformative innovation in the digital art market by conferring verifiable ownership and scarcity upon digital works. While the global crypto art market has experienced substantial growth, consumer adoption in China remains nascent and poorly understood, constrained by regulatory particularities and limited empirical investigation.

**Methods:**

This study applies the extended Unified Theory of Acceptance and Use of Technology (UTAUT2) to examine the determinants of crypto art adoption among Chinese consumers. Drawing on survey data from 342 respondents with prior arttransaction experience and employing structural equation modeling (SEM), we test a comprehensive model incorporating performance expectancy, effort expectancy, social influence, facilitating conditions, hedonic motivation, price value, and habit as predictors of behavioral intention and usage behavior.

**Results:**

The results indicate that performance expectancy, facilitating conditions, hedonic motivation, price value, and habit significantly and positively influence consumers' behavioral intention, whereas effort expectancy and social influence do not. Furthermore, facilitating conditions, habit, and behavioral intention directly predict actual usage behavior. The model explains 74.6% of the variance in behavioral intention and 67.3% in usage behavior.

**Discussion:**

The non-significance of social influence is consistent with the personal and private nature of crypto art consumption in China's distinctive regulatory environment. These findings yield four contributions. First, the study extends the UTAUT2 framework to the under-researched domain of crypto art. Second, the nonsignificance of social influence suggests that its predictive power may be contingent on the social visibility of consumption, the maturity of normative expectations, and the institutional framing of the technology rather than on cultural context alone. Third, we identify a dual-layer structure of habit in super-app ecosystems, distinguishing between domain-specific habit and ecosystem-level habit. Fourth, we observe boundary conditions of effort expectancy in high-involvement consumption, wherein consumers' willingness to invest cognitive resources appears to attenuate the role of perceived ease of use. These contributions extend UTAUT2 to the underresearched context of digital art consumption and offer actionable insights for platform developers, artists, and policymakers.

## 1 Introduction

The advent of blockchain technology has catalyzed a paradigm shift in the creation, ownership, and exchange of digital artifacts, giving rise to crypto art—a novel category of digital artwork whose authenticity, provenance, and scarcity are cryptographically guaranteed through non-fungible tokens (NFTs) (Franceschet et al., 2021). Unlike conventional digital files that can be duplicated indefinitely at zero marginal cost, tokenized artworks confer exclusive ownership rights immutably recorded on distributed ledgers, thereby resolving the fundamental challenge of digital scarcity that had historically precluded digital art from functioning as a collectible asset class (Joselit, 2021). This technological innovation has reconfigured established norms of art production, distribution, and valuation, opening new frontiers for creative expression and cultural consumption in the digital economy (Murray et al., 2023).

The market significance of crypto art became globally visible in March 2021, when the digital artist Beeple's NFT collage everydays: The first 5,000 days was auctioned at Christie's for USD 69.3 million, positioning crypto art as a legitimate category within the high-value art market (Joselit, 2021). Since then, the NFT market has expanded rapidly: total NFT trading volume reached approximately USD 24.7 billion in 2022, with art and collectibles constituting a substantial share (Tong, 2022; Technavio, 2022). In China, despite regulatory constraints on cryptocurrency transactions, the market for digital collectibles (shuzi cangpin)—the domestic analog of NFTs operating on permissioned consortium blockchains—experienced explosive growth, with platforms such as Jingtan (Alibaba) and Huanhe (Tencent) attracting millions of users (Zhang, 2023; Andy and Tian, 2022). Nevertheless, following initial speculative exuberance, the Chinese market entered a phase of consolidation and regulatory recalibration, characterized by stricter oversight of secondary trading and an emphasis on cultural digitization over financial speculation (Wang, 2023). Recent policy developments have further clarified the boundaries of compliant digital collectible operations. In April 2022, the China Internet Finance Association, the China Banking Association, and the Securities Association of China jointly issued an initiative on preventing the financial risks of non-fungible tokens, urging platforms to implement real-name authentication, prohibit cryptocurrency settlement and secondary-market speculation, and align digital collectible content with officially endorsed cultural narratives (China Internet Finance Association, 2022). In parallel, the national strategy for cultural digitalization has positioned digital assets within the state-sanctioned cultural economy (Guo et al., 2024; Li and Zhan, 2024). These developments underscore the need for rigorous empirical research on the factors shaping consumer engagement with crypto art in China's distinctive institutional environment.

Despite the growing market prominence of crypto art, scholarly understanding of consumer behavior in this domain remains underdeveloped. Extant research on crypto art and NFTs has predominantly concentrated on technical infrastructure (Regner et al., 2019; Muthe et al., 2020), legal and copyright implications (Guadamuz, 2021; de Sousa, 2022), market dynamics and price formation (Horky et al., 2022; Mekacher et al., 2022), and philosophical or aesthetic critiques (Lyubchenko, 2022; Radermecker and Ginsburgh, 2023). A smaller but expanding body of work has examined consumer motivations in NFT markets, identifying factors such as perceived uniqueness, social status, investment potential, and community belonging as drivers of purchase intention (Vishnu Prasad et al., 2024; Fortagne and Lis, 2024; Griffiths et al., 2024). However, these studies have largely been conducted in Western contexts and have not systematically applied established technology acceptance frameworks to model the multi-dimensional determinants of adoption behavior. Furthermore, the Chinese crypto art market exhibits structural characteristics—governmental restrictions on cryptocurrency payments, the predominance of consortium-blockchain-based digital collectibles, and culturally specific aesthetic preferences—that limit the generalizability of findings from Western samples (Wang, 2023; Tian, 2024). Consequently, a theoretically grounded, contextually sensitive investigation of Chinese consumers' adoption of crypto art is both timely and warranted.

The Unified Theory of Acceptance and Use of Technology 2 (UTAUT2), developed by Venkatesh et al. (2012), offers a comprehensive and empirically robust framework for examining consumer technology adoption. UTAUT2 synthesizes eight foundational theories of technology acceptance—including the Technology Acceptance Model (TAM), Innovation Diffusion Theory (IDT), and the Theory of Planned Behavior (TPB)—into a unified model comprising seven core constructs: performance expectancy, effort expectancy, social influence, facilitating conditions, hedonic motivation, price value, and habit (Venkatesh et al., 2012, 2003). The model has demonstrated superior explanatory power relative to its predecessor theories across diverse consumer domains, including mobile banking (Baptista and Oliveira, 2015), telemedicine (Schmitz et al., 2022), wearable health technologies (Talukder et al., 2020), e-sports (Ong et al., 2023), mobile payment (Migliore et al., 2022), and short-video platform engagement (Liu et al., 2024), consistently explaining 60–75% of variance in behavioral intention. The applicability of UTAUT2 to hedonic, consumption-oriented technologies makes it particularly well-suited for investigating the adoption of crypto art, which straddles the boundary between technological innovation and aesthetic experience.

This study contributes to the literature in four principal ways. First, it extends the UTAUT2 model to the novel and rapidly evolving domain of crypto art, testing its applicability beyond the utilitarian and organizational contexts in which it has predominantly been validated, and identifies the boundary conditions of effort expectancy in high-involvement consumption contexts. Second, it provides the first quantitative examination of crypto art adoption among Chinese consumers, offering insights into how China's unique regulatory architecture and digital consumption culture shape adoption dynamics. Third, it empirically tests the role of social influence—a core yet contested construct in technology acceptance research—within the distinctive social and regulatory context of Chinese crypto art consumption, revealing that its predictive power is contingent on the social visibility of the consumption behavior, the maturity of normative expectations, and the institutional framing of the technology. Fourth, by identifying the dual-layer structure of habit in super-app ecosystems and the relative importance of different adoption determinants, the study generates actionable guidance for platform designers, digital artists, market intermediaries, and policymakers navigating this emerging sector. The remainder of this paper is organized as follows: Section 2 reviews the theoretical background and develops the research hypotheses. Section 3 describes the methodology. Section 4 presents the results. Section 5 discusses the findings in relation to prior literature and outlines theoretical and practical implications. Section 6 concludes with limitations and directions for future research.

## 2 Theoretical background and hypothesis development

### 2.1 Crypto art: concept, market, and research landscape

Crypto art refers to digital artworks whose ownership and authenticity are certified by NFTs—unique, indivisible cryptographic tokens recorded on a blockchain (Franceschet et al., 2021; Diaz et al., 2023). The technological architecture of blockchain ensures that each tokenized artwork possesses a verifiable provenance trail, recording every transaction from the work's initial minting through all subsequent transfers of ownership (Valeonti et al., 2021). This innovation addresses a fundamental limitation of pre-blockchain digital art: the impossibility of distinguishing an original from a perfect copy. By introducing artificial scarcity and authenticated ownership into the digital realm, crypto art has enabled the emergence of a global market for digitally native creative works that functions analogously to, yet remains distinct from, the traditional fine art market (Guan et al., 2024; Xia et al., 2024).

The creation and circulation of crypto art diverge from conventional art world practices in several important respects. Artists can mint and list works directly on NFT marketplaces without the intermediation of galleries, dealers, or auction houses, substantially reducing barriers to market entry and enabling creators from non-traditional backgrounds to monetize their work (Sharma et al., 2022). Smart contracts can be programmed to automatically execute royalty payments to artists upon each secondary sale, potentially addressing long-standing concerns about artists' exclusion from the appreciation in their works' value (Zhao, 2021). Transactions are typically settled in cryptocurrency, affording participants a degree of pseudonymity while simultaneously generating a fully transparent, publicly auditable record of market activity (Zarifis and Castro, 2022). For collectors, crypto art offers novel forms of engagement, including the ability to display works in virtual environments, participate in decentralized governance of artist communities, and trade assets across global, continuously operating markets (Zarifis and Castro, 2022).

The Chinese crypto art ecosystem has evolved along a distinctive trajectory shaped by the government's restrictive stance on cryptocurrency speculation (Cai et al., 2021). Beginning in 2021, Chinese authorities implemented a series of measures prohibiting financial institutions from facilitating cryptocurrency transactions, effectively severing the link between domestic digital collectible platforms and the global crypto economy (Wang, 2023). In response, major Chinese internet enterprises developed domestic digital collectible platforms (He et al., 2022)—such as Alibaba's Jingtan and Tencent's Huanhe—that operate on permissioned consortium blockchains, use fiat currency (RMB) for transactions, and restrict or prohibit secondary market trading (Zhang, 2023; Andy and Tian, 2022). This model, characterized in official discourse as ‘definancialization' (qu jinrong hua), prioritizes the cultural and collectible dimensions of digital assets while suppressing speculative and investment-oriented behaviors (Tian, 2024). Recent regulatory frameworks have further codified these principles, requiring platforms to implement real-name authentication, restrict resale mechanisms, and align content with officially endorsed cultural narratives (Guo et al., 2024; Li and Zhan, 2024). Consequently, the Chinese market for crypto art diverges from global norms in terms of payment methods, trading liquidity, platform governance, and the cultural framing of digital ownership, rendering it a theoretically rich case for examining technology adoption under institutional constraints. In this paper, we therefore use the term “crypto art” as an umbrella concept that encompasses both NFTs on public blockchains and China's domestically regulated digital collectibles (shuzi cangpin); the latter constitute the concrete objects that our Chinese respondents could adopt and transact. We position these digital collectibles within the broader crypto art and NFT literature because they share the same underlying tokenization logic—cryptographically guaranteed scarcity and verifiable ownership—while differing in blockchain infrastructure, settlement currency, and secondary-market regulation.

Scholarly research on crypto art has proliferated across multiple disciplines. In computer science and engineering, studies have addressed blockchain infrastructure (Regner et al., 2019), NFT standards (Dowling, 2022; Ferone and Porta, 2022), and environmental sustainability concerns associated with energy-intensive consensus mechanisms (Truby et al., 2022). Legal scholarship has examined intellectual property implications, including the distinction between token ownership and copyright ownership (Guadamuz, 2021; de Sousa, 2022), and the enforceability of resale royalty rights (Zhao, 2021). Economic analyses have investigated price determinants (Horky et al., 2022; Mekacher et al., 2022), market efficiency and bubble behavior (Wang et al., 2022), and the role of rarity and visual attributes in value formation (Nadini et al., 2021). Within the humanities, scholars have debated the artistic merit and cultural significance of crypto art (Calvo, 2024), with some framing it as a digital avant-garde (Lyubchenko, 2022) and others questioning its aesthetic value relative to established artistic traditions (Radermecker and Ginsburgh, 2023). However, empirical research on the end-users of crypto art—consumers, collectors, and enthusiasts—remains strikingly scarce. The limited existing studies in this vein have identified factors including perceived financial value, social status signaling, technological curiosity, and community affiliation as drivers of NFT purchase intention (Vishnu Prasad et al., 2024; Fortagne and Lis, 2024; Griffiths et al., 2024), yet none has employed a comprehensive, theoretically integrated model of technology acceptance to systematically analyze the interplay of utilitarian, hedonic, social, and habitual determinants of consumer behavior. The present study addresses this gap by deploying the UTAUT2 framework in the Chinese crypto art context.

### 2.2 The UTAUT2 framework

The Unified Theory of Acceptance and Use of Technology (UTAUT), originally proposed by Venkatesh et al. (2003), integrated constructs from eight prominent models of technology acceptance—including TAM, IDT, the Theory of Reasoned Action (TRA), TPB, the Motivation Model (MM), the combined TAM-TPB model, the Model of PC Utilization (MPCU), and Social Cognitive Theory (SCT)—into a parsimonious framework comprising four direct determinants of behavioral intention and usage: performance expectancy, effort expectancy, social influence, and facilitating conditions, moderated by gender, age, experience, and voluntariness of use (Venkatesh et al., 2003). Empirical testing demonstrated that UTAUT explained approximately 70% of the variance in behavioral intention, outperforming each of its constituent models individually (Venkatesh et al., 2003).

Recognizing that UTAUT had been developed primarily for organizational settings and did not adequately capture the distinctive attributes of consumer technology adoption—particularly the roles of intrinsic motivation, cost considerations, and routinized behavior—Venkatesh et al. (2012) extended the model to produce UTAUT2. This revised framework incorporated three additional constructs: hedonic motivation (the pleasure or enjoyment derived from technology use), price value (consumers' cognitive trade-off between the perceived benefits of a technology and its monetary cost), and habit (the extent to which individuals perform behaviors automatically as a result of learning). The moderating roles of gender, age, and experience were retained (Venkatesh et al., 2012). UTAUT2 has since been validated in a wide range of consumer contexts, including mobile payment adoption (Migliore et al., 2022), mobile health applications (Schmitz et al., 2022), e-learning platforms (Moorthy et al., 2019), autonomous delivery services (Kapser and Abdelrahman, 2020), social media marketing (Chatterjee and Kar, 2020), short-video commerce (Liu et al., 2024), and metaverse engagement (Arpaci et al., 2022; Dwivedi et al., 2023). A systematic review by Tamilmani et al. (2021) confirmed that UTAUT2 consistently achieves high explanatory power (*R*^2^ values typically ranging from 0.60 to 0.75 for behavioral intention) and demonstrates robust cross-contextual validity.

The selection of UTAUT2 for the present study is motivated by several considerations. Crypto art consumption is fundamentally a technology-mediated behavior (Tan et al., 2024): it requires interaction with blockchain-based platforms, digital wallets, and token marketplaces, and consumer decisions are shaped by assessments of platform usability, transactional efficiency, enjoyment, cost, social norms, and accumulated experience—all constructs central to the UTAUT2 framework. Moreover, crypto art straddles the boundary between utilitarian technology (as a tool for collecting and transacting) and hedonic technology (as a source of aesthetic pleasure and entertainment), making the dual emphasis of UTAUT2 on extrinsic and intrinsic motivational drivers particularly appropriate. Finally, the model's demonstrated capacity to accommodate contextual adaptations—through the selective inclusion or exclusion of constructs and the introduction of domain-specific modifications—aligns with the exploratory character of this study, which represents the first application of a unified technology acceptance framework to the crypto art domain.

### 2.3 Conceptual model and hypotheses

In adapting UTAUT2 for the crypto art context, we retained all seven core constructs specified in the original model (Venkatesh et al., 2012), including social influence. Social influence refers to the degree to which individuals perceive that important others (family, friends, colleagues, and—in the contemporary Chinese digital environment—key opinion leaders and online community members) believe they should adopt a given technology (Venkatesh et al., 2003). The inclusion of social influence in the present model is theoretically motivated by the extensive literature documenting the potency of social and normative pressures in shaping Chinese consumer behavior, particularly in technology-mediated consumption contexts. In China's highly social digital ecosystem, where platforms such as WeChat, Douyin, and Xiaohongshu facilitate intense peer-to-peer information sharing, social endorsement, and community-based consumption (Migliore et al., 2022; Chen et al., 2024), social influence has been shown to significantly predict adoption intentions across domains including mobile payment (Migliore et al., 2022), social commerce (Xiao et al., 2023), and live-streaming shopping (Zhang et al., 2024). Within the crypto art domain specifically, social dynamics—such as community affiliation, artist following, and status signaling through collection display—have been identified as potentially relevant motivational factors (Vishnu Prasad et al., 2024; Fortagne and Lis, 2024). However, given the relatively low public visibility of crypto art in China, the private and personalized nature of digital art appreciation, and the regulatory environment that discourages conspicuous speculation, it remains an open empirical question whether social influence exerts a significant effect on adoption intention in this specific context. Accordingly, we include social influence as a construct in our model and test its effect empirically rather than excluding it *a priori*.

The moderating variables of gender, age, and experience specified in the original UTAUT2 model were not included in this initial exploratory application, as our primary objective was to establish the baseline direct effects of the core constructs in a novel domain. The resulting conceptual model, comprising nine constructs and ten hypothesized relationships, is depicted in Figure 1 and elaborated below.

**Figure 1:**
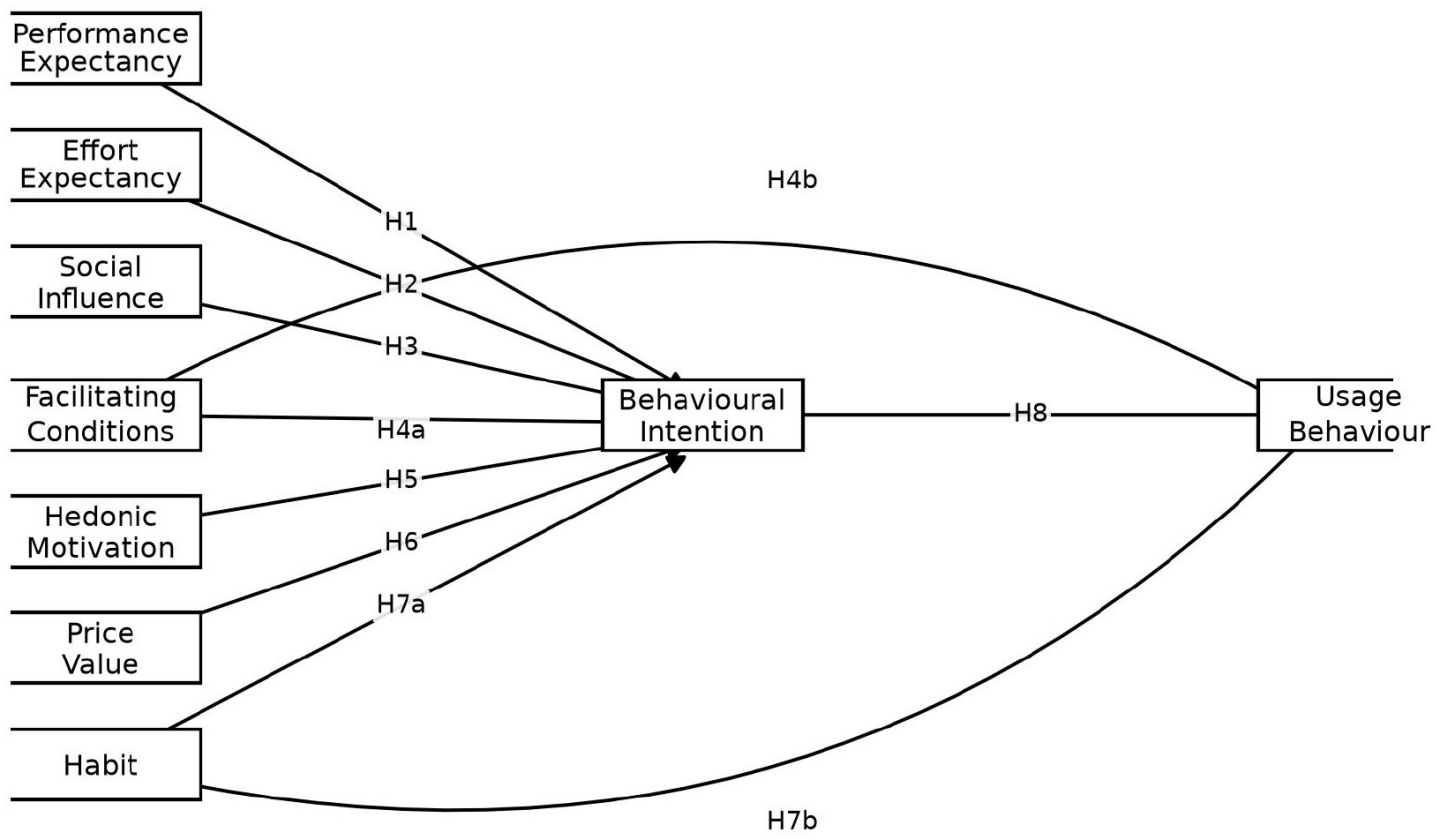
Proposed conceptual model.

#### 2.3.1 Performance expectancy

Performance expectancy captures the degree to which individuals believe that using a technology will enhance their performance in relevant activities (Venkatesh et al., 2003). In organizational contexts, this construct has consistently emerged as the strongest predictor of behavioral intention (Venkatesh et al., 2012, 2003). In the crypto art domain, performance expectancy encompasses consumers' beliefs that engaging with crypto art will improve the efficiency, convenience, and outcomes of their art-related activities—including discovery, acquisition, collection management, and potential economic returns. Consumers who perceive crypto art as offering advantages over traditional art forms in terms of accessibility, transaction speed, portfolio diversification, and provenance verification are more likely to form positive adoption intentions (Franceschet et al., 2021; Horky et al., 2022). Accordingly, we hypothesize:

H1. Performance expectancy positively influences consumers' behavioral intention to adopt crypto art.

#### 2.3.2 Effort expectancy

Effort expectancy denotes the perceived ease associated with using a technology (Venkatesh et al., 2003). Technologies perceived as intuitive and requiring minimal cognitive or operational effort are more readily adopted, particularly in consumer contexts where users face low switching costs and abundant alternatives (Xu et al., 2012). In the crypto art context, effort expectancy relates to the perceived simplicity of navigating NFT platforms, setting up digital wallets, comprehending token standards, and completing transactions. Although the technical infrastructure of crypto art involves complex underlying processes (blockchain consensus, cryptographic key management, smart contract execution), consumer-facing interfaces may abstract away this complexity. We hypothesize:

H2. Effort expectancy positively influences consumers' behavioral intention to adopt crypto art.

#### 2.3.3 Social influence

Social influence refers to the degree to which individuals perceive that important others believe they should adopt a given technology (Venkatesh et al., 2003). In the Chinese consumer context, social influence operates through multiple channels: interpersonal influence from family and friends, community-based influence from online interest groups, and parasocial influence from key opinion leaders (KOLs) and content creators on platforms such as Douyin, Xiaohongshu, and WeChat (Migliore et al., 2022; Zhang et al., 2024). Prior UTAUT2 research in Chinese settings has documented significant effects of social influence on adoption intentions for mobile payment systems (Migliore et al., 2022), social commerce platforms (Xiao et al., 2023), and live-streaming shopping (Zhang et al., 2024). In the crypto art domain, social dynamics—such as community affiliation on platforms like Discord or domestic alternatives, artist-fan relationships, status signaling through collection display on social media, and peer recommendations—have been identified as potentially relevant motivational factors in qualitative studies of NFT collectors (Vishnu Prasad et al., 2024; Fortagne and Lis, 2024). However, crypto art consumption in China also exhibits characteristics that may attenuate the role of social influence. The regulatory environment discourages conspicuous displays of speculative activity, digital art appreciation is often a private, aesthetically driven experience, and the relatively niche status of crypto art means that normative expectations within consumers' immediate social circles may be weak or absent. Given these countervailing considerations, the direction and magnitude of social influence's effect on crypto art adoption intention in China is theoretically ambiguous and warrants direct empirical investigation. We hypothesize:

H3. Social influence positively influences consumers' behavioral intention to adopt crypto art.

#### 2.3.4 Facilitating conditions

Facilitating conditions reflect individuals' perceptions of the resources, knowledge, and support available to enable technology use (Venkatesh et al., 2003). In the UTAUT2 model, facilitating conditions are hypothesized to influence both behavioral intention and actual usage behavior (Venkatesh et al., 2012). For crypto art adoption, facilitating conditions encompass access to compatible devices (smartphones, computers), reliable internet connectivity, availability of user-friendly platforms with Chinese-language interfaces, adequate customer support, accessible educational resources about blockchain and NFTs, and a regulatory environment that provides sufficient legal clarity for consumers to transact with confidence (Zarifis and Castro, 2022; Li and Zhan, 2024). When these infrastructural and support elements are perceived to be in place, consumers are not only more inclined to form adoption intentions but also more capable of translating those intentions into actual behavior. We hypothesize:

H4a. Facilitating conditions positively influence consumers' behavioral intention to adopt crypto art.
H4b. Facilitating conditions positively influence consumers' actual usage behavior of crypto art.

#### 2.3.5 Hedonic motivation

Hedonic motivation refers to the pleasure, enjoyment, or fun derived from using a technology (Venkatesh et al., 2012). In consumer contexts, hedonic motivation has been identified as a particularly salient determinant of technology acceptance, often rivaling or exceeding utilitarian considerations in predictive strength (Shaw and Sergueeva, 2019; Schomakers et al., 2022). Crypto art consumption is intrinsically an aesthetic and experiential activity: consumers browse digital galleries, appreciate visual creativity, discover emerging artists, and derive satisfaction from curating personal collections. The multimedia features of crypto art platforms—including high-resolution artwork display, interactive visualization tools, gamified engagement mechanisms, and social sharing capabilities—further amplify the hedonic dimension of the experience. When crypto art-related activities are perceived as enjoyable and entertaining, consumers' willingness to engage increases. We hypothesize:

H5. Hedonic motivation positively influences consumers' behavioral intention to adopt crypto art.

#### 2.3.6 Price value

Price value captures consumers' cognitive trade-off between the perceived benefits of using a technology and the monetary costs associated with its use (Venkatesh et al., 2012). Unlike in organizational settings, where the employing organization typically bears technology costs, consumers in voluntary-use contexts directly incur financial expenditures, making price-value assessments a critical determinant of adoption decisions (Shao and Siponen, 2011). For crypto art, the cost-benefit calculus is multifaceted: consumers evaluate not only the purchase price of NFT artworks but also transaction fees (gas fees on public blockchains or service fees on consortium chains), the costs of acquiring and securing cryptocurrency or digital wallets, and the opportunity costs of allocating financial resources to digital rather than physical art assets. In the Chinese market, the prevalent 1VN distribution model—in which a single artwork is issued as multiple limited-edition digital tokens at relatively accessible price points—further shapes price-value perceptions by lowering entry barriers (Zhang, 2023; Andy and Tian, 2022). When consumers perceive that the benefits derived from crypto art (aesthetic enjoyment, collection utility, potential appreciation) exceed its financial costs, their adoption intention strengthens. We hypothesize:

H6. Price value positively influences consumers' behavioral intention to adopt crypto art.

#### 2.3.7 Habit

Habit is defined as the extent to which individuals tend to perform behaviors automatically as a consequence of prior learning and repeated experience (Limayem et al., 2007). Habitual behavior is characterized by automaticity, reduced cognitive deliberation, and resistance to change. Within the UTAUT2 framework, habit is hypothesized to influence both behavioral intention and usage behavior directly, and its effect on behavior is posited to operate partially independently of conscious intention (Venkatesh et al., 2012). In the crypto art context, consumers who have developed routines around browsing NFT marketplaces, following artist communities, monitoring market trends, and collecting digital works may come to engage in these activities as a matter of course rather than as the product of deliberate decision-making at each instance. The self-reinforcing cycle of habitual engagement is particularly relevant to crypto art in the Chinese context, where major platforms are embedded within the super-app ecosystems of Alibaba and Tencent. Platforms such as Jingtan are accessible through Alipay's mini-program infrastructure, meaning that consumer engagement with digital collectibles may be partially driven by the pre-existing, highly routinized habit of opening Alipay for daily transactions—a behavior performed by hundreds of millions of Chinese users multiple times per day (Chen and Zhou, 2024). This ecosystem integration may amplify the role of habit beyond what would be observed in standalone platform contexts. We hypothesize:

H7a. Habit positively influences consumers' behavioral intention to adopt crypto art.
H7b. Habit positively influences consumers' actual usage behavior of crypto art.

#### 2.3.8 Behavioral intention

Behavioral intention, rooted in the Theory of Reasoned Action (Ajzen, 1980) and the Theory of Planned Behavior (Ajzen, 1985), represents an individual's conscious plan or motivation to perform a specified behavior. Across the technology acceptance literature, behavioral intention has been robustly established as the most proximal and powerful predictor of actual technology use (Venkatesh et al., 2012, 2003; Davis, 1989). In this study, behavioral intention captures consumers' expressed willingness to engage in crypto art-related activities—including browsing, purchasing, collecting, and recommending crypto artworks—in the foreseeable future. Consistent with the cumulative evidence from UTAUT2 research, we hypothesize:

H8. Behavioral intention positively influences consumers' actual usage behavior of crypto art.

## 3 Method

### 3.1 Instrument development

All measurement items were adapted from previously validated scales, principally the UTAUT2 instrument of Venkatesh et al. (2012) and the UTAUT instrument of Venkatesh et al. (2003), with wording modified to fit the crypto art context. Each construct was operationalized using three to four items rated on a Likert-type scale. To ensure content validity, the adapted items were reviewed by two experts in consumer behavior and digital technology and pilot-tested with a small group of respondents, after which ambiguous items were revised prior to full administration. Social influence was measured using three items adapted from the original UTAUT2 scale. Table 1 presents the full set of latent variables and their observed indicators.

**Table 1:** Definition of latent and observed variables.

Latent variable	Observed variable	Content of variable
Performance expectancy	PE1	I find crypto art useful in my daily life
	P*E2*	Using crypto art increases my chances of realizing an art collection
	PE3	Using crypto art helps me collect art faster
	PE4	Using crypto art increases my art collection
Effort expectancy	EE1	Learning how to use crypto art is easy for me
	EE2	My interaction with crypto art is clear and understandable
	EE3	I find crypto art easy to use
	EE4	It is easy for me to become skilful at crypto art
Social influence	SI1	People who are important to me think that I should use crypto art
	SI2	People who influence my behavior think that I should use crypto art
	SI3	People whose opinions that I value prefer that I use crypto art
Facilitating conditions	FC1	I have the resources necessary to use crypto art
	FC2	I have the knowledge necessary to use crypto art
	FC3	Crypto art is compatible with other technologies I use
	FC4	I can get help from others when I have difficulties using crypto art
Hedonic motivation	HM1	Using crypto art Internet is fun
	HM2	Using crypto art is enjoyable
	HM3	Using crypto art is very entertaining
Price value	PV1	Crypto art is reasonably priced
	PV2	Crypto art is a good value for the money
	PV3	At the current price, crypto art provides a good value
Habit	HT1	The use of crypto art has become a habit for me
	HT2	I am addicted to using crypto art
	HT3	I must use crypto art
Behavioral intention	BI1	I will always try to use crypto art in the future
	BI2	I intend to continue using crypto art in my daily life
	BI3	I would recommend crypto art to others
	BI4	I plan to become a crypto artist
Usage behavior	UB1	How often I use crypto art
	UB2	How often I buy crypto art

### 3.2 Data collection

Data were collected through a structured survey administered to Chinese consumers over a one-month period (March 2025), employing a dual-mode (online and offline) recruitment strategy. Respondents were recruited primarily online through social media platforms (WeChat and Weibo), digital-art and collectible enthusiast communities, and snowball referrals, supplemented by offline recruitment at university art exhibitions; participation was voluntary and respondents resided across multiple regions of China. The target population comprised adults aged 18 years and older with an interest in or exposure to crypto art. Screening questions were used to identify respondents with prior experience in art-related transactions (encompassing creation, exhibition, auction, or collecting), which served as a proxy for basic art-market familiarity. This screening criterion did not require prior experience with crypto art, NFTs, or digital collectibles; rather, it ensured that participants possessed sufficient familiarity with art-market concepts to provide meaningful responses concerning crypto art adoption. Respondents who did not meet this criterion were excluded from the survey.

To enhance response quality, participants were first presented with an informational video explaining the fundamental concepts of crypto art and NFTs, including their technological basis, market mechanisms, and differences from traditional art forms. To ensure the neutrality of the video content, its script was restricted to technical descriptions of blockchain, NFTs, and transaction processes, and did not contain any value judgments, promotional language, or normative claims about the desirability of crypto art participation, thereby minimizing potential priming effects on respondents (Podsakoff et al., 2012). Following this orientation, respondents completed the questionnaire anonymously. To incentivize conscientious participation, respondents who submitted high-quality, verifiable responses received a modest monetary compensation. Survey quality was monitored by tracking completion times and responses to embedded attention-check items. We conducted an *a priori* power analysis for the structural model, which indicated that a minimum of approximately 200 respondents was required to detect medium-sized path coefficients with adequate statistical power (Hair et al., 2010; Kline, 2023). Following these recommendations, we targeted a sample size of 200–400 respondents. The final valid dataset comprised 342 complete responses (response rate: 79.9%), meeting the recommended threshold for SEM analysis with the specified number of latent constructs and indicators. Table 2 summarizes the demographic characteristics of the sample.

**Table 2:** Respondent characteristics.

Characteristic	Items	Frequency	Percentage	Mean represents	Standard deviation
Gender	Male	207	60.5%	1.39	0.49
	Female	135	39.5%		
Year of birth	1995–2010	174	50.9%	1.56	0.64
	1980–1995	147	42.9%		
	1965–1980	18	5.3%		
	1945–1965	3	0.9%		
Usage frequency	Used occasionally	42	12.3%	3.61	0.85
	Used more often	92	26.9%		
	Used frequently	167	48.8%		
	Used everyday	41	12.0%		

### 3.3 Common method bias assessment

Because data were collected from a single source using self-report measures, common method bias (CMB) represented a potential validity threat. Several procedural remedies were implemented during questionnaire design: items measuring different constructs were placed on separate pages to create psychological separation; respondents were assured of response anonymity and instructed that there were no right or wrong answers; and item wording was refined to minimize ambiguity and social desirability bias.

Statistically, we employed a multi-method approach to CMB assessment. First, Harman's single-factor test (Harman, 1976) was conducted by entering all measurement items into an unrotated exploratory factor analysis. The Kaiser-Meyer-Olkin (KMO) measure was 0.962 (*p* < 0.001), confirming sampling adequacy for factor analysis; we note that KMO is a diagnostic of sampling adequacy rather than a direct test of common method bias. Nine factors with eigenvalues exceeding 1.0 were extracted, collectively accounting for 80.98% of total variance. The first factor explained 13.92% of variance—well below the 50% threshold that would indicate a dominant single factor—providing an initial, non-definitive indication that common method bias did not materially distort the study's findings.

To further strengthen the CMB assessment, we employed the unmeasured latent method construct (ULMC) technique (Podsakoff et al., 2003; Liang et al., 2007). A common method factor was introduced into the CFA model, with all measurement items allowed to load simultaneously on their substantive constructs and on a single method factor, after which the substantive and method-factor loadings were compared. The variance attributable to the method factor was 11.7%, substantially below the 25% threshold identified by Williams et al. (2010) as indicative of serious method bias. Moreover, a comparison of the standardized path coefficients between the structural model with and without the method factor revealed that all key path coefficient differences were less than 0.03, indicating that CMB did not substantively alter the pattern or significance of the hypothesized relationships. Collectively, these procedural and statistical safeguards—while diagnostic rather than conclusive—provide reasonable confidence that common method bias does not pose a major threat to the validity of our findings.

### 3.4 Analytical approach

Structural equation modeling (SEM) was conducted using IBM SPSS Statistics version 27.0 (IBM Corp., Armonk, NY, USA) and IBM SPSS AMOS version 28 (IBM Corp., Armonk, NY, USA) software. Following the two-step analytical procedure recommended by Anderson and Gerbing (Anderson and Gerbing, 1988), we first evaluated the measurement model through confirmatory factor analysis (CFA) to establish the reliability, convergent validity, and discriminant validity of the latent constructs. Subsequently, the structural model was assessed to test the hypothesized relationships. Model fit was evaluated using a combination of absolute and incremental fit indices: the chi-square to degrees of freedom ratio (χ^2^/df), Adjusted Goodness of Fit Index (AGFI), Comparative Fit Index (CFI), Tucker–Lewis Index (TLI), and Root Mean Square Error of Approximation (RMSEA), with thresholds derived from Hu and Bentler (Hu and Bentler, 1999). Data screening confirmed that skewness (all values < |2|) and kurtosis (all values < |6|) were within acceptable ranges for maximum likelihood estimation (Collier, 2020).

## 4 Results

### 4.1 Measurement model evaluation

The measurement model demonstrated good fit to the data (Table 3): χ^2^/df = 1.870, GFI = 0.893, AGFI = 0.861, CFI = 0.968, TLI = 0.962, NFI = 0.935, and RMSEA = 0.051, all meeting the conventional thresholds recommended by Hu and Bentler (1999), Bagozzi and Yi (1988).

**Table 3:** Model fit indices.

Fit indices	CMIN/DF	GFI	AGFI	CFI	TLI	NFI	RMSEA
Recommended values	< 3	>0.80	>0.80	>0.90	>0.90	>0.90	< 0.08
Measurement model	1.870	0.893	0.861	0.968	0.962	0.935	0.051
Research model	1.863	0.892	0.862	0.968	0.962	0.934	0.050

CMIN, chi-square minimum fit function; DF, degrees of freedom; CMIN/DF, chi-square to degrees of freedom ratio; AGFI, adjusted goodness of fit index; CFI, comparative fit index; TLI, Tucker–Lewis index; RMSEA, root mean square error of approximation.

Table 4 reports the standardized factor loadings, descriptive statistics, and reliability estimates. All standardized factor loadings exceeded 0.70 (ranging from 0.724 to 0.914). Composite reliability (CR) ranged from 0.831 to 0.922 and Cronbach's alpha ranged from 0.825 to 0.922, both exceeding the 0.70 threshold, and all average variance extracted (AVE) values exceeded 0.50 (ranging from 0.609 to 0.797), confirming convergent validity (Fornell and Larcker, 1981).

**Table 4:** Descriptive statistics, reliability, and convergent validity.

Construct	Item	Factor loading	Kurtosis	Skewness	Cronbach α	AVE	CR
PE	PE1	0.800	−0.462	−0.098	0.862	0.609	0.862
	PE2	0.799	−0.63	−0.002			
	PE3	0.797	−0.701	0.12			
	PE4	0.724	−0.498	0.305			
EE	EE1	0.813	−0.465	−0.381	0.903	0.698	0.902
	EE2	0.862	−0.774	0.718			
	EE3	0.839	−0.839	0.956			
	EE4	0.827	−0.639	0.209			
SI	SI1	0.874	−0.620	0.410	0.911	0.769	0.909
	SI2	0.898	−0.710	0.550	0.911	0.769	0.909
	SI3	0.859	−0.580	0.330	0.911	0.769	0.909
FC	FC1	0.868	−0.982	1.151	0.919	0.740	0.919
	FC2	0.882	−0.992	1.115			
	FC3	0.844	−0.65	0.188			
	FC4	0.846	−0.827	0.682			
HM	HM1	0.848	−0.872	1.14	0.865	0.682	0.865
	HM2	0.810	−0.856	1.291			
	HM3	0.819	−0.815	0.534			
PV	PV1	0.855	−0.688	0.159	0.903	0.759	0.904
	PV2	0.851	−0.429	−0.107			
	PV3	0.907	−0.762	0.935			
HT	HT1	0.881	−0.74	0.493	0.922	0.797	0.922
	HT2	0.912	−0.694	0.223			
	HT3	0.885	−0.525	−0.103			
BI	BI1	0.819	−0.627	0.74	0.874	0.699	0.874
	BI2	0.841	−0.739	0.823			
	BI3	0.847	−0.544	0.153			
UB	UB1	0.914	−0.88	0.276	0.825	0.713	0.831
	UB2	0.768	−0.822	0.277			

St. Est., standardized estimates; α, Cronbach α > 0.7; AVE, average variance extracted > 0.5; CR, composite reliability > 0.7.

Kurtosis ≤ ± 6; Skewness ≤ ± 2.

Discriminant validity was assessed using both the Fornell–Larcker criterion and the heterotrait–monotrait (HTMT) ratio of correlations (Henseler et al., 2015) (Table 5). The square root of each construct's AVE (shown on the diagonal) exceeded its correlations with all other constructs, and all HTMT values were below the conservative 0.85 threshold (the highest being 0.749), supporting discriminant validity.

**Table 5:** Discriminant validity (Fornell–Larcker criterion and HTMT ratios).

Construct	PE	EE	SI	FC	BI	HM	PV	HT	UB
PE	0.781	0.593	0.720	0.655	0.672	0.63	0.597	0.583	0.542
EE	0.681^***^	0.835	0.680	0.731	0.597	0.598	0.551	0.645	0.546
SI	0.663^***^	0.624^***^	0.877	0.740	0.720	0.700	0.660	0.710	0.670
FC	0.741^***^	0.802^***^	0.682^***^	0.86	0.729	0.705	0.63	0.715	0.666
BI	0.778^***^	0.675^***^	0.658^***^	0.811^***^	0.836	0.749	0.743	0.73	0.674
HM	0.735^***^	0.680^***^	0.631^***^	0.788^***^	0.862^***^	0.826	0.716	0.729	0.609
PV	0.680^***^	0.612^***^	0.598^***^	0.681^***^	0.833^***^	0.807^***^	0.871	0.708	0.558
HT	0.662^***^	0.710^***^	0.642^***^	0.777^***^	0.817^***^	0.816^***^	0.776^***^	0.893	0.709
UB	0.642^***^	0.624^***^	0.607^***^	0.757^***^	0.803^***^	0.719^***^	0.652^***^	0.799^***^	0.844

The diagonal is the square root of the AVE value, below the diagonal is the Fornell–Larcker criterion, and above the diagonal is the Heterotrait-Monotrait ratio (HTMT). The symbol ^***^ indicates significance at *p* ≤ 0.001 (two-tailed).

### 4.2 Structural model and hypothesis testing

Table 6 presents the standardized path coefficients and the hypothesis-testing results. Of the ten hypothesized relationships, eight were supported and two were not. Performance expectancy (β = 0.196, *p* < 0.01), facilitating conditions (β = 0.254, *p* < 0.01), hedonic motivation (β = 0.232, *p* < 0.05), price value (β = 0.271, *p* < 0.001), and habit (β = 0.168, *p* < 0.05) significantly predicted behavioral intention, whereas effort expectancy (β = −0.108, *p* = 0.095) and social influence (β = 0.058, *p* = 0.265) did not. Facilitating conditions (β = 0.208, *p* < 0.05), habit (β = 0.372, *p* < 0.001), and behavioral intention (β = 0.318, *p* < 0.01) significantly predicted usage behavior. The structural model explained 74.6% of the variance in behavioral intention and 67.3% of the variance in usage behavior (Figure 2).

**Table 6:** Structural model: path coefficients and hypothesis testing results.

Hypothesis	Path			β	S.E.	C.R.	*p*	Decision
H1	PE	→	BI	0.196	0.075	3.052	0.002^**^	Supported
H2	EE	→	BI	−0.108	0.046	−1.672	0.095 n.s.	Not Supported
H3	SI	→	BI	0.058	0.052	1.115	0.265 n.s.	Not Supported
H4a	FC	→	BI	0.254	0.059	3.077	0.002^**^	Supported
H4b	FC	→	UB	0.208	0.075	2.550	0.011^*^	Supported
H5	HM	→	BI	0.232	0.073	2.499	0.012^*^	Supported
H6	PV	→	BI	0.271	0.055	3.824	^***^	Supported
H7a	HT	→	BI	0.168	0.045	2.230	0.026^*^	Supported
H7b	HT	→	UB	0.372	0.066	4.395	^***^	Supported
H8	BI	→	UB	0.318	0.126	3.263	0.001^**^	Supported

^***^Significant at *p* ≤ 0.001 (two-tailed).

^**^Significant at *p* < 0.010 (two-tailed).

^*^Significant at *p* < 0.050 (two-tailed).

n.s., not significant.

**Figure 2:**
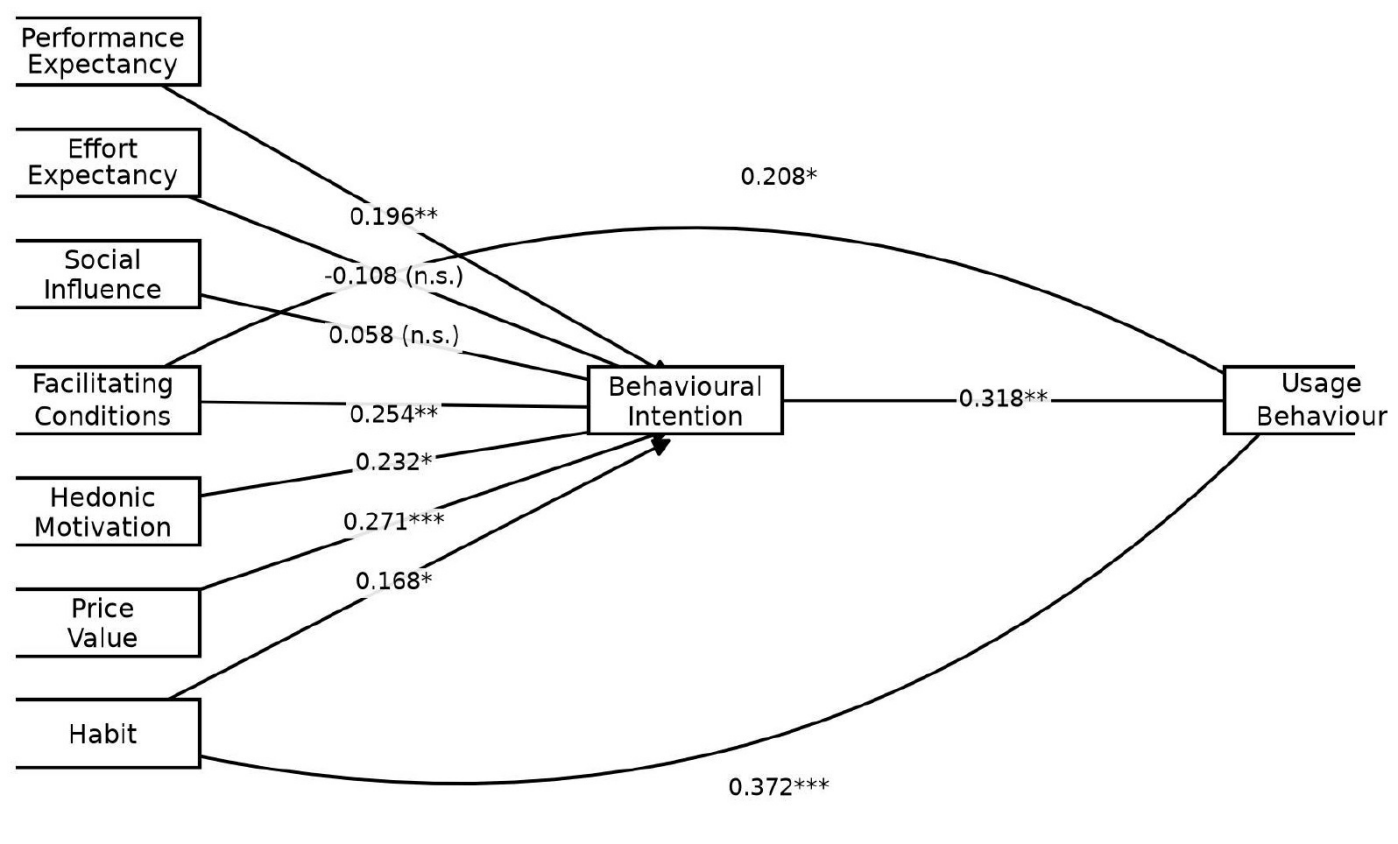
Summary of the structural model results. Standardized path coefficients are displayed on each path. ***p < 0.001, **p < 0.01, *p < 0.05.

## 5 Discussion

This study set out to identify the factors influencing Chinese consumers' adoption of crypto art by applying and empirically testing an extended UTAUT2 model. The findings offer a nuanced account of the determinants of both behavioral intention and actual usage behavior in this emerging domain. Overall, the model demonstrates strong explanatory power (*R*^2^ = 0.746 for intention; *R*^2^ = 0.673 for behavior), confirming the applicability of the UTAUT2 framework to the crypto art context and extending its empirical reach beyond the organizational and utilitarian consumer technologies that have dominated prior validation efforts (Venkatesh et al., 2012; Tamilmani et al., 2021). In the following subsections, we interpret each finding in light of the existing literature, discuss broader theoretical and practical implications, and situate the results within the distinctive institutional and cultural landscape of the Chinese digital art market.

### 5.1 Interpretation of key findings

Before proceeding to the detailed interpretation of individual findings, we note a methodological consideration relevant to the interpretation of the overall pattern of results. The informational video used during data collection to establish respondents‘ baseline understanding of crypto art may have subtly influenced their subsequent survey responses, particularly those related to hedonic motivation and habit. Although the video script was restricted to technical descriptions and contained no value judgments or promotional content (see Section 3.2), it is possible that exposure to a professionally produced orientation stimulus enhanced respondents' interest in and positive affect toward crypto art, thereby modestly amplifying the observed effects of experiential and habitual constructs. It is worth noting, however, that the pattern and relative magnitudes of the path coefficients are broadly consistent with prior UTAUT2 research in unrelated consumer technology domains (Venkatesh et al., 2012; Tamilmani et al., 2021), which mitigates concern that video-induced priming accounts for the core findings. We encourage readers to interpret the results with this methodological nuance in mind and return to this consideration in Section 6.1.

#### 5.1.1 Performance expectancy and effort expectancy

The significant positive effect of performance expectancy on behavioral intention (H1 supported, β = 0.196, *p* < 0.01) corroborates the foundational premise of UTAUT2 that consumers' technology adoption decisions are substantially driven by expected utilitarian benefits (Venkatesh et al., 2012, 2003). Consumers who perceive crypto art as enhancing the efficiency, accessibility, and outcomes of their art-related activities—for example, by providing faster access to diverse artworks, reducing intermediary costs, or enabling more transparent provenance verification—are more inclined to form positive adoption intentions. This finding aligns with UTAUT2-based research in domains such as cloud storage adoption (Song et al., 2020), mobile health applications (Uncovska et al., 2023), and 5G technology acceptance (Mustafa et al., 2022), where performance expectancy has consistently emerged as a significant predictor. The result suggests that, despite the aesthetic and experiential character of crypto art, utilitarian considerations retain substantial influence over consumer decision-making.

In contrast, effort expectancy did not significantly predict behavioral intention (H2 not supported, β = −0.108, *p* = 0.095). While this finding diverges from the original UTAUT2 specification (Venkatesh et al., 2012), it is consistent with a growing body of evidence indicating that effort expectancy may exert diminishing influence as consumer technologies mature and user interfaces become increasingly intuitive (Martinez and McAndrews, 2023; Suo et al., 2022). We propose three complementary explanations, grounded in both the broader technology acceptance literature and the specific characteristics of the Chinese digital consumption environment.

First, from a task-technology fit (TTF) perspective (Goodhue and Thompson, 1995), crypto art consumption constitutes a high-involvement activity in which consumers engage for aesthetic gratification, identity expression, and potential financial returns. In such high-involvement consumption domains, consumers are typically willing to invest significant time and cognitive resources to master the requisite tools and platforms, because the anticipated hedonic and utilitarian value of successful participation outweighs the learning costs. This ‘motivated proficiency' mechanism has been documented in other high-involvement digital consumption contexts, such as PC gaming and cryptocurrency trading, where ease-of-use concerns are subordinated to outcome expectations and intrinsic motivation (Alalwan, 2020). In effect, when the perceived value of the consumption object is sufficiently high, consumers become relatively insensitive to the effort required—they do not expect the experience to be ‘easy', and may even interpret complexity as a signal of authenticity and exclusivity.

Second, the major domestic crypto art platforms—Jingtan, Huanhe, and their competitors—have invested substantially in user experience design, offering streamlined mobile applications that abstract away blockchain complexity and integrate seamlessly with consumers' existing digital routines. Chinese consumers, who are among the world's most experienced users of mobile payment systems and digital platforms (Migliore et al., 2022), may possess a level of digital literacy that attenuates the impact of effort-related concerns in technology adoption decisions. Third, the non-significant finding may partially reflect a self-selection effect, compounded by the sample's demographic composition. As detailed in Section 5.2, our sample is predominantly young (93.9% of respondents born between 1980 and 2010) and highly educated (85.4% holding a bachelor's degree or above). This demographic profile is associated with higher baseline digital literacy, which may further suppress the salience of effort expectancy in shaping adoption intentions. Consumers who are sufficiently motivated to explore crypto art and possess strong digital skills may have already overcome initial learning barriers, rendering ease-of-use considerations less salient in their ongoing adoption calculus. This interpretation is consistent with Venkatesh et al. (2003) argument that effort expectancy is most consequential during initial technology exposure and diminishes with accumulated experience. We return to the implications of the sample's demographic composition for the generalizability of this finding in Section 5.2.

#### 5.1.2 The non-significance of social influence (H3)

A noteworthy finding of this study is the non-significant effect of social influence on behavioral intention (H3 not supported, β = 0.058, *p* = 0.265). This result is striking given the extensive evidence documenting the potency of social influence in Chinese consumer technology adoption contexts (Migliore et al., 2022; Xiao et al., 2023; Zhang et al., 2024), and warrants careful theoretical interpretation.

We propose three interlocking explanations for this finding. First, crypto art consumption in China is characterized by a high degree of personalization and privacy. Unlike social commerce or live-streaming shopping—domains in which social influence operates through visible, real-time peer behavior and community endorsement (Zhang et al., 2024)—crypto art appreciation and collection are predominantly solitary, aesthetically driven activities. Digital artworks are typically viewed on personal devices, collections are managed in private digital wallets, and the regulatory environment discourages conspicuous displays of digital asset ownership that could signal status or wealth (Wang, 2023; Tian, 2024). In this context, the normative and informational social mechanisms that drive adoption in more socially visible consumption domains may be attenuated or absent.

Second, crypto art has not yet attained the level of mainstream social visibility in China that would be necessary for social influence to crystallize as a meaningful adoption driver. Unlike mobile payment, short-video platforms, or e-commerce—technologies around which robust social norms, shared usage expectations, and peer endorsement networks have developed—crypto art remains a niche interest. Most Chinese consumers have limited exposure to crypto art through their immediate social circles, and the topic rarely features in everyday social discourse (Guo et al., 2024; Li and Zhan, 2024). In the absence of established social norms and visible peer behavior, the informational and normative pathways through which social influence typically operates lack the necessary substrate.

Third, the regulatory framing of digital collectibles in China as cultural products rather than financial assets (Tian, 2024) may further suppress the social dynamics—such as status competition, conspicuous consumption, and fear of missing out (FOMO)—that have been identified as drivers of NFT adoption in less regulated markets (Vishnu Prasad et al., 2024; Fortagne and Lis, 2024). The ‘definancialization' policy framework actively discourages the speculative and socially performative dimensions of crypto art engagement, encouraging instead a more contemplative, culturally oriented mode of consumption that is less susceptible to social influence. This finding suggests that the role of social influence in technology adoption is not culturally invariant but is instead contingent on domain-specific characteristics—including the social visibility of the consumption behavior, the maturity of normative expectations within the relevant community, and the institutional and regulatory framing of the technology. We discuss the theoretical implications of this finding further in Section 5.2.

#### 5.1.3 Facilitating conditions

Facilitating conditions emerged as a significant predictor of both behavioral intention (H4a supported, β = 0.254, *p* < 0.01) and actual usage behavior (H4b supported, β = 0.208, *p* < 0.05). This dual-pathway influence is consistent with the theoretical rationale of UTAUT2 (Venkatesh et al., 2012) and replicates findings from mobile banking (Baptista and Oliveira, 2015), e-sports (Ong et al., 2023), and mobile payment (Migliore et al., 2022) research. The significant effect on behavioral intention suggests that consumers evaluate the adequacy of infrastructural and support resources when forming their attitudinal disposition toward crypto art. More importantly, the direct effect on usage behavior—unmediated by intention—indicates that even consumers with favorable attitudes may be thwarted in their attempts to engage with crypto art if practical barriers are not adequately addressed. This finding carries particular relevance in the Chinese context, where the regulatory separation of domestic digital collectible platforms from the global cryptocurrency ecosystem introduces idiosyncratic infrastructural requirements—such as RMB-denominated transactions, domestic identity verification procedures, and compliance with platform-specific terms of service—that may constitute obstacles for less technologically proficient consumers (Wang, 2023).

#### 5.1.4 Hedonic motivation

Hedonic motivation significantly and positively predicted behavioral intention (H5 supported, β = 0.232, *p* < 0.05), underscoring the experiential and pleasure-driven character of crypto art consumption. This finding aligns with the broader UTAUT2 literature demonstrating the salience of intrinsic motivation in consumer technology adoption (Shaw and Sergueeva, 2019; Schomakers et al., 2022; Eneizan et al., 2019) and resonates with qualitative evidence that NFT collectors derive substantial gratification from the aesthetic appreciation, discovery, and curation dimensions of their engagement (Vishnu Prasad et al., 2024; Fortagne and Lis, 2024). The significance of hedonic motivation carries implications for platform design: features that enhance the entertainment value and sensory richness of the crypto art experience—such as high-resolution artwork display, virtual gallery environments, gamified collection mechanics, and social sharing functionalities—are likely to amplify consumer engagement. Furthermore, the finding suggests that crypto art platforms should not be conceptualized solely as transactional marketplaces but rather as experiential environments that cater to consumers' aesthetic sensibilities and desire for enjoyable interaction.

#### 5.1.5 Price value

Price value exerted the strongest influence on behavioral intention among all predictors (H6 supported, β = 0.271, *p* < 0.001), highlighting the centrality of cost-benefit calculations in consumers' adoption decisions. This finding is consistent with UTAUT2 research in price-sensitive consumer technology domains (Shao and Siponen, 2011; Zhou, 2008) and aligns with economic analyses documenting the importance of price accessibility in NFT market participation (Horky et al., 2022; Mekacher et al., 2022). The prominence of price value in the Chinese context may be amplified by the prevailing 1VN distribution model, whereby artists issue multiple limited-edition tokens for a single artwork at accessible price points, effectively lowering the entry barrier relative to traditional art collecting (Zhang, 2023; Andy and Tian, 2022). Consumers appear to engage in deliberate assessments of whether the bundle of benefits conferred by crypto art ownership—encompassing aesthetic enjoyment, collection utility, social signaling, and potential financial appreciation—justifies the monetary expenditure. Platforms and creators should therefore attend carefully to pricing transparency, the perceived fairness of fee structures, and the communication of value propositions to consumers.

#### 5.1.6 Habit and the Chinese super-app ecosystem

Habit significantly predicted both behavioral intention (H7a supported, β = 0.168, *p* < 0.05) and, more substantially, actual usage behavior (H7b supported, β = 0.372, *p* < 0.001). The magnitude of habit's direct effect on behavior—exceeding that of behavioral intention (β = 0.318)—is a noteworthy finding that merits careful interpretation in light of the distinctive characteristics of the Chinese digital ecosystem.

This pattern is consistent with the theoretical proposition, advanced by Limayem et al. (2007) and empirically corroborated in UTAUT2 research across mobile banking (Baptista and Oliveira, 2015), mobile visual search (Meng et al., 2024), and e-sports (Ong et al., 2023), that habitual behavior can partially supplant conscious intention as a driver of technology use. As consumers accumulate repeated, satisfying experiences with crypto art platforms, their engagement may become increasingly routinized—browsing marketplaces, monitoring artist communities, and acquiring new works become patterned behaviors performed with diminished conscious deliberation.

Beyond this general mechanism, we argue that the particularly strong effect of habit observed in this study reflects a structural feature of the Chinese digital marketplace: the integration of digital collectible platforms into the country's dominant super-app ecosystems. Jingtan (Alibaba's digital collectible platform) is accessible through Alipay's mini-program infrastructure, which is embedded within an application that hundreds of millions of Chinese consumers use multiple times daily for payments, transportation, food delivery, and financial management (Chen and Zhou, 2024). Similarly, Huanhe and other platforms are integrated within Tencent's ecosystem, accessible via WeChat mini-programs. This architectural feature means that consumer engagement with crypto art is not necessarily the product of a discrete, deliberate decision to ‘visit a crypto art platform'; rather, it may arise as a by-product of pre-existing, deeply routinized behavioral patterns—such as the habit of opening Alipay to check transactions or browsing WeChat for content. The platform ecosystem effectively ‘piggybacks' crypto art engagement onto high-frequency habitual behaviors, thereby amplifying the role of habit in driving usage.

This interpretation constitutes a significant theoretical contribution: it suggests that in super-app-dominated digital ecosystems, the habit construct in UTAUT2 may operate through two distinct mechanisms—domain-specific habit (repeated, satisfying interactions with the crypto art platform itself) and ecosystem-level habit (routinized engagement with the super-app within which the platform is embedded). The latter mechanism has been under-explored in UTAUT2 research, which has predominantly been conducted in contexts where the focal technology operates as a standalone application. Future research should examine whether and how super-app embedding moderates the role of habit in technology adoption across different domains and cultural contexts.

#### 5.1.7 Behavioral intention

Consistent with the accumulated evidence from the technology acceptance tradition (Venkatesh et al., 2012, 2003; Davis, 1989), behavioral intention significantly and positively predicted usage behavior (H8 supported, β = 0.318, *p* < 0.01). This finding reaffirms the role of behavioral intention as a proximal determinant of technology use, even in a domain as novel and affectively charged as crypto art. The significant path from intention to behavior, combined with the robust explanatory power of the antecedent constructs, suggests that the UTAUT2 model provides a coherent and empirically supported account of the cognitive and motivational processes through which Chinese consumers form and enact decisions to engage with crypto art.

### 5.2 Theoretical implications

This study makes several contributions to the theoretical literature on technology acceptance and digital consumption. First, it extends the empirical reach of the UTAUT2 model to the domain of crypto art—a context that differs substantially from the utilitarian, productivity-oriented technologies in which UTAUT2 has predominantly been validated. The high explanatory power achieved (*R*^2^ = 0.746 for behavioral intention) attests to the model's robustness and versatility, while the differential significance and effect sizes of individual constructs illuminate context-specific adoption dynamics that generic model application would obscure. In particular, the primacy of price value and hedonic motivation over performance expectancy departs from the pattern typically observed in organizational technology adoption studies (Venkatesh et al., 2003) and underscores the importance of calibrating theoretical models to the specific characteristics of the technology and user population under investigation.

Second, the non-significance of social influence—a core UTAUT2 construct that has demonstrated robust predictive power in numerous Chinese consumer technology contexts (Migliore et al., 2022; Xiao et al., 2023; Zhang et al., 2024)—represents a theoretically generative finding that we advance as a proposition for future research. It suggests that the relevance of social influence is not a simple function of the cultural context (i.e., ‘social influence matters in collectivist cultures') but is instead jointly determined by the social visibility of the consumption behavior, the maturity of normative expectations within the relevant domain, and the institutional framing of the technology. In domains where consumption is private, norms are nascent, and regulatory frameworks discourage conspicuous engagement, the mechanisms through which social influence typically operates may be structurally constrained. We emphasize that this explanation is a contextual interpretation grounded in the Chinese institutional environment rather than a directly tested mechanism. This insight contributes to a more nuanced, contingency-based understanding of social influence in technology acceptance, moving beyond the simplistic assumption that social influence is uniformly potent in collectivist cultural settings.

Third, the finding that habit's direct effect on behavior exceeds that of behavioral intention, interpreted through the lens of China's super-app ecosystem architecture, suggests—as a proposition for future research—that ecosystem-level habit may operate as a distinct mechanism from domain-specific habit. This distinction may have relevance beyond the crypto art context, applying to any technology whose adoption is mediated by integration into a high-frequency platform ecosystem. Future theoretical work on UTAUT2 should consider explicitly modeling the multi-level nature of habit in platform-mediated consumption environments.

Fourth, the study contributes to the nascent empirical literature on consumer behavior in crypto art and NFT markets. While prior work has predominantly adopted qualitative, theoretical, or narrowly behavioral approaches (Vishnu Prasad et al., 2024; Fortagne and Lis, 2024; Griffiths et al., 2024), this research provides a quantitative, model-driven account that situates crypto art adoption within a nomological network of established behavioral determinants. By demonstrating that consumers' engagement with crypto art is shaped by a combination of utilitarian expectations, hedonic gratifications, cost-benefit assessments, facilitating conditions, and habituated routines—but not by social influence or effort-related considerations—the study offers a more comprehensive and theoretically integrated picture than has previously been available.

Finally, it is important to acknowledge that the demographic composition of our sample—predominantly young (93.9% of respondents born between 1980 and 2010), highly educated (85.4% with bachelor's degrees or above), and urban—may condition several of the findings reported above. The non-significance of effort expectancy, in particular, may partially reflect the high digital literacy of this demographic group, who are likely to find blockchain-based platforms less cognitively demanding than would older, less educated, or rural consumers. Similarly, the attenuated effect of social influence may reflect this cohort's greater comfort with individualized, digitally mediated consumption practices that are less dependent on traditional social referents. Consequently, the generalizability of these findings to older age cohorts, consumers with lower educational attainment, or those in non-urban regions should be approached with appropriate caution. We return to this important limitation in Section 6.1 and outline directions for future research that would address this generalizability concern through multi-group and cross-demographic comparative designs.

### 5.3 Practical implications

The implications below are offered as reasoned, evidence-informed suggestions rather than as direct empirical consequences of the tested model; none of the specific strategies described was itself manipulated or tested in the present design.

#### 5.3.1 Implications for platform operators

The findings of this study carry actionable implications for crypto art platform developers and operators in the Chinese market. The significance of hedonic motivation and habit suggests that investments in user experience design—encompassing intuitive navigation, visually engaging interfaces, gamification features, personalization algorithms, and community-building tools—are likely to yield returns in the form of enhanced user engagement and retention. Specifically, platforms should leverage the super-app integration advantage: by optimizing the discoverability and accessibility of digital collectible features within Alipay and WeChat mini-program environments, platforms can capitalize on consumers' pre-existing app-usage habits to drive engagement. For instance, platforms could implement ‘habit-stacking' strategies—positioning digital collectible browsing as a natural extension of consumers' daily Alipay or WeChat routines through strategically timed notifications, homepage widgets, and contextually relevant recommendations.

The primacy of price value (the strongest predictor of behavioral intention) underscores the importance of transparent, accessible, and strategically differentiated pricing. The prevalent 1VN distribution model appears to effectively lower barriers to entry, but platforms can further optimize their pricing architecture through: (a) tiered rarity systems that create clear, communicable value differentiation across price points; (b) artist-signed limited editions that command premium pricing while preserving mass-market accessibility through standard editions; (c) transparent disclosure of all platform fees and blockchain-related costs to build consumer trust in the fairness of the pricing structure; and (d) time-limited promotional pricing for new user acquisition, calibrated to convert initial trial into habitual engagement.

The significant dual-pathway effect of facilitating conditions—on both intention and behavior—highlights the critical importance of user education and support infrastructure. Platforms should invest in: (a) comprehensive, Chinese-language educational resources (interactive tutorials, video guides, glossaries of blockchain terminology) that lower knowledge barriers for new users; (b) responsive, accessible customer support channels integrated within the super-app environment; (c) streamlined identity verification and wallet setup procedures that minimize friction during onboarding; and (d) clear, prominently displayed information about consumers' legal rights, platform policies, and data privacy protections, which is particularly important given the regulatory complexities of the Chinese digital collectible market (Guo et al., 2024; Li and Zhan, 2024).

#### 5.3.2 Strategies for artists and content creators

For digital artists and content creators operating in or entering the Chinese market, the findings offer guidance on audience development and commercial strategy. The significance of hedonic motivation implies that artworks and collections that deliver aesthetic pleasure, entertainment value, and emotional resonance are more likely to attract consumer interest than those positioned solely as speculative or investment assets. Artists should invest in the narrative and experiential dimensions of their work: crafting compelling ‘story-driven' collection concepts, providing behind-the-scenes content that deepens collectors' emotional connection to the creative process, and designing interactive or multimedia elements that extend the aesthetic experience beyond static visual appreciation.

The importance of habit for driving usage behavior suggests that artists should adopt consistent, rhythm-based engagement strategies to cultivate habitual following among collectors. Practical tactics include: (a) serialized collection releases at regular, predictable intervals to establish “appointment-based” collector behavior; (b) time-limited “airdrop” events that reward habitual followers with exclusive content; (c) community engagement rituals—such as weekly artist AMAs (Ask Me Anything), work-in-progress sharing, or collector voting on future creative directions—that create recurring interaction patterns; and (d) loyalty or ‘completion' rewards that incentivize collectors to acquire full series. Such strategies are consistent with the finding that habitual engagement, once established, can sustain usage behavior partially independently of conscious purchase intention.

The prominence of price value indicates that artists should carefully calibrate their pricing and edition strategies to align with the value expectations of Chinese consumer segments. The 1VN model provides a useful baseline, but artists can differentiate their offerings through hybrid edition structures that combine accessible “entry-level” tokens with premium, highly scarce “signature” editions. Transparent communication about edition sizes, pricing rationale, and the artist's long-term commitment to the collection's value can further strengthen consumer confidence in the fairness of the price-value proposition.

#### 5.3.3 Insights for policymakers and regulators

For policymakers and regulators, the study provides evidence that consumer adoption of crypto art in China is driven by a heterogeneous mix of motivational factors—utilitarian expectations, aesthetic enjoyment, perceived value for money, and routinized platform engagement—rather than by speculative or socially contagious impulses alone. The non-significance of social influence is particularly instructive in this regard: it suggests that the current regulatory approach, which emphasizes the cultural rather than financial dimensions of digital collectibles and discourages conspicuous, socially driven speculative behavior, may be effectively channeling consumer engagement toward intrinsic (hedonic and utilitarian) motivations. While this pattern is consistent with—rather than a direct test of—the “definancialization” policy framework, it suggests that maintaining clear regulatory boundaries between cultural digital assets and speculative financial instruments can foster a consumer ecosystem in which adoption is driven by sustainable, product-intrinsic motivations rather than by socially amplified speculative dynamics.

At the same time, the significance of facilitating conditions underscores the importance of providing regulatory clarity and accessible consumer protection mechanisms. Policymakers should consider: (a) developing clear, publicly accessible guidelines on the legal status of digital collectibles, consumer rights in platform disputes, and intellectual property protections for both creators and collectors (Guo et al., 2024); (b) establishing standards for platform transparency regarding fee structures, edition authenticity, and secondary market restrictions; and (c) supporting public education initiatives that enhance consumers' blockchain and digital asset literacy, thereby strengthening facilitating conditions across a broader demographic spectrum. Such measures would not only protect consumers but also contribute to the long-term legitimacy and sustainability of the digital collectible market as a component of China's cultural digital economy (Li and Zhan, 2024; Tian, 2024).

## 6 Conclusion

This study has provided the first quantitative examination of the factors influencing Chinese consumers' adoption of crypto art, employing an extended UTAUT2 framework to model the interplay of performance expectancy, effort expectancy, social influence, facilitating conditions, hedonic motivation, price value, habit, and behavioral intention. The results, derived from structural equation modeling of survey data from 342 respondents, yield several key conclusions. Empirically, crypto art adoption in China is primarily driven by price value, facilitating conditions, hedonic motivation, performance expectancy, and habit, with the model explaining 74.6% of variance in behavioral intention, while effort expectancy and social influence do not exert significant effects. Theoretically, the study offers four contributions to the technology acceptance literature. First, it extends the UTAUT2 framework to the novel domain of crypto art, confirming its applicability beyond utilitarian and organizational contexts. Second, the results suggest—as a proposition for future research—that the predictive power of social influence may depend not merely on cultural context but on the social visibility of consumption, the maturity of normative expectations, and the institutional framing of the technology. Third, we identify a dual-layer structure of habit in super-app ecosystems, distinguishing between domain-specific habit and ecosystem-level habit as distinct mechanisms driving technology use. Fourth, we observe the boundary conditions of effort expectancy in high-involvement consumption, wherein consumers' willingness to invest cognitive resources appears to attenuate the role of perceived ease of use. These findings confirm the applicability of UTAUT2 to the crypto art domain while revealing context-specific dynamics that enrich the theoretical framework and inform stakeholder practice.

### 6.1 Limitations and future research

Several limitations of the present study should be acknowledged, each suggesting directions for future inquiry. First, the reliance on cross-sectional, self-reported survey data introduces the possibility of common method variance and precludes causal inference regarding the temporal dynamics of adoption. Although we employed both procedural remedies and multiple statistical safeguards—including Harman's single-factor test and the unmeasured latent method construct technique—to assess and mitigate CMB, longitudinal designs that track consumers' crypto art engagement over time would substantially strengthen causal claims and illuminate the developmental trajectories of adoption behavior, from initial awareness through trial, regular use, and potentially disengagement.

Second, the sample, while adequate for the SEM analyses conducted, was drawn from a relatively narrow demographic band: predominantly young (93.9% of respondents born between 1980 and 2010), highly educated (85.4% with bachelor's degrees or above), and urban. As noted in Section 5.2, this demographic composition may condition several of our key findings—particularly the non-significance of effort expectancy (potentially reflecting high digital literacy in this cohort) and the attenuated effect of social influence (potentially reflecting this group's comfort with individualized digital consumption practices). The generalizability of these findings to older age cohorts, consumers with lower educational attainment, or those in non-urban regions should therefore be approached with appropriate caution. Future research should endeavor to recruit larger, more demographically diverse samples, potentially through probability-based sampling frames, and conduct multi-group analyses to examine whether the structural relationships identified in this study are invariant across age cohorts, education levels, income strata, and geographic regions. Such comparative designs would be invaluable for establishing the boundary conditions of our findings and identifying demographic segments for whom different adoption determinants may be differentially salient.

Third, the present study excluded the moderating variables—gender, age, and experience—specified in the original UTAUT2 model, focusing instead on establishing baseline direct effects in a novel domain. Subsequent research should investigate whether and how these demographic and experiential factors moderate the relationships observed here, as such moderation analyses could reveal theoretically and practically important heterogeneity in adoption mechanisms across consumer segments. Fourth, the model did not incorporate constructs that may be particularly relevant to crypto art adoption, such as perceived risk (financial, privacy, technological), trust in blockchain technology and platform intermediaries, aesthetic sensitivity, social identity and community affiliation, and environmental concern regarding blockchain energy consumption. Future extensions of the UTAUT2 framework that integrate such domain-specific constructs could further enhance explanatory power and theoretical specificity.

Fifth, although we took care to ensure the neutrality of the informational video used during data collection—its script was restricted to technical descriptions and contained no value judgments or promotional language—the use of any orienting stimulus may introduce subtle priming effects that could influence subsequent survey responses (Podsakoff et al., 2012). Future research could explore alternative designs, such as text-only information sheets or control-group comparisons with and without video intervention, to assess the magnitude of any such effects.

Sixth, and more broadly, the study was conducted within a single national context characterized by distinctive regulatory and cultural features that may limit generalizability to other settings. Cross-national comparative research that examines how institutional environments—including regulatory frameworks, payment infrastructures, and cultural attitudes toward digital ownership—moderate the determinants of crypto art adoption would be valuable for developing a more globally comprehensive understanding of this phenomenon. Finally, as blockchain technology, NFT standards, and platform business models continue to evolve rapidly, the determinants of consumer adoption identified in this study may themselves be subject to change. Ongoing programmatic research that tracks the crypto art ecosystem as it matures will be essential for maintaining the ecological validity and practical relevance of scholarly knowledge in this domain.

## Data Availability

The raw data supporting the conclusions of this article will be made available by the authors, without undue reservation.

## References

[B1] Ajzen I. (1980). Understanding Attitudes and Predicting Social Behavior. Englewood Cliffs, NJ: Prentice-Hall.

[B2] Ajzen I. (1985). “From intentions to actions: a theory of planned behavior,” in Action Control: From Cognition to Behavior, eds. J. Kuhl and J. Beckmann (Berlin: Springer), 11–39. doi: 10.1007/978-3-642-69746-3_2

[B3] Alalwan A. A. (2020). Mobile food ordering apps: an empirical study of the factors affecting customer e-satisfaction and continued intention to reuse. Int. J. Inf. Manag. 50, 28–44. doi: 10.1016/j.ijinfomgt.2019.04.008

[B4] Anderson J. C. Gerbing D. W. (1988). Structural equation modeling in practice: a review and recommended two-step approach. Psychol. Bull. 103, 411–423. doi: 10.1037/0033-2909.103.3.411

[B5] Andy S. Tian Z. (2022). China's NFT Market in 2022: The Most Comprehensive Analysis Guide. Enlybee. Available online at: https://www.enlybee.com/the-most-comprehensive-report-to-chinas-nft-market/ (Accessed July 15, 2025).

[B6] Arpaci I. Karatas K. Kusci I. Al-Emran M. (2022). Understanding the social sustainability of the metaverse by integrating UTAUT2 and big five personality traits. Technol. Soc. 71:102120. doi: 10.1016/j.techsoc.2022.102120

[B7] Bagozzi R. P. Yi Y. (1988). On the evaluation of structural equation models. J. Acad. Mark. Sci. 16, 74–94. doi: 10.1007/BF02723327

[B8] Baptista G. Oliveira T. (2015). Understanding mobile banking: the unified theory of acceptance and use of technology combined with cultural moderators. Comput. Hum. Behav. 50, 418–430. doi: 10.1016/j.chb.2015.04.024

[B9] Cai L. Sun Y. Zheng Z. Xiao J. Qiu W. (2021). Blockchain in China. Commun. ACM 64, 88–93. doi: 10.1145/3481627

[B10] Calvo P. (2024). Cryptoart: ethical challenges of the NFT revolution. Humanit. Soc. Sci. Commun. 11, 1–10. doi: 10.1057/s41599-024-02872-2

[B11] Chatterjee S. Kar A. K. (2020). Why do small and medium enterprises use social media marketing and what is the impact. Int. J. Inf. Manag. 53:102103. doi: 10.1016/j.ijinfomgt.2020.102103

[B12] Chen L. Wang S. Zhou Y. (2024). Social influence in the age of social media: examining the role of peer networks in Chinese consumers technology adoption. Inf. Technol. People 37, 456–473.

[B13] Chen Y. Zhou T. (2024). The rise of super-apps in China: Alipay and WeChat as digital infrastructure. New Media Soc. 26, 1510–1528.

[B14] China Internet Finance Association China Banking Association, and Securities Association of China.. (2022). Initiative on Preventing the Financial Risks of Non-fungible Tokens. Beijing: China Internet Finance Association. (in Chinese)

[B15] Collier J. (2020). Applied Structural Equation Modeling Using AMOS: Basic to Advanced Techniques. New York, NY: Routledge. doi: 10.4324/9781003018414

[B16] Davis F. D. (1989). Perceived usefulness, perceived ease of use, and user acceptance of information technology. MIS Q. 13, 319–340. doi: 10.2307/249008 23455082

[B17] de Sousa F. M. (2022). Token-art system and the new international art market: the impacts of NFT technology and the legal aspects involved. J. Law Mark. Innov. 1, 97–115.

[B18] Diaz M. A. V. Gutierrez J. M. Rodriguez L. F. (2023). “Exploring the fusion of technology and art: unveiling the historical landscape of art NFTs,” in 2023 IEEE Colombian Caribbean Conference (C3) (Barranquilla: IEEE), 1–5. doi: 10.1109/C358072.2023.10436222

[B19] Dowling M. (2022). Fertile LAND: pricing non-fungible tokens. Financ. Res. Lett. 44:102096. doi: 10.1016/j.frl.2021.102096

[B20] Dwivedi Y. K. Hughes L. Baabdullah A. M. Ribeiro-Navarrete S. Giannakis M. Al-Debei M. M. . (2023). Metaverse marketing: how the metaverse will shape the future of consumer research and practice. Psychol. Mark. 40, 750–776. doi: 10.1002/mar.21767

[B21] Eneizan B. Alsaad A. Abdelbaset A. Obaid A. (2019). Customer acceptance of mobile marketing in Jordan: an extended UTAUT2 model with trust and service quality. Int. J. Eng. Bus. Manag. 11, 1–11. doi: 10.1177/1847979019889484

[B22] Ferone A. Porta A. D. (2022). A blockchain-based infection tracing and notification system by non-fungible tokens. Comput. Commun. 192, 66–74. doi: 10.1016/j.comcom.2022.05.027 35669083 PMC9159782

[B23] Fornell C. Larcker D. F. (1981). Evaluating structural equation models with unobservable variables and measurement error. J. Mark. Res. 18, 39–50. doi: 10.1177/002224378101800104

[B24] Fortagne M. A. Lis B. (2024). Determinants of the purchase intention of non-fungible token collectibles. J. Consum. Behav. 23, 1032–1049. doi: 10.1002/cb.2264

[B25] Franceschet M. Colavizza G. Smith T. Finucane B. Ostachowski M. L. Scalet S. . (2021). Crypto art: a decentralized view. Leonardo 54, 402–405. doi: 10.1162/leon_a_02003

[B26] Goodhue D. L. Thompson R. L. (1995). Task-technology fit and individual performance. MIS Q. 19, 213–236. doi: 10.2307/249689

[B27] Griffiths P. Costa C. J. Fernandes Crespo N. (2024). Behind the bubble: exploring the motivations of NFT buyers. Comput. Hum. Behav. 158:108307. doi: 10.1016/j.chb.2024.108307

[B28] Guadamuz A. (2021). The treachery of images: non-fungible tokens and copyright. J. Intellect. Prop. Law Pract. 16, 1367–1385. doi: 10.1093/jiplp/jpab152

[B29] Guan M. Y. Li J. Wang T. Zhang W. (2024). From digital art to crypto art: the evolution of art brought by NFT. Int. J. Hum.–Comput. Interact. 40, 1–20.

[B30] Guo B. Liu J. Zhang W. Chen X. (2024). Legal regulation of non-fungible token digital collection trading in China. J. Econ. Law 1, 14–19. doi: 10.62517/jel.202414303

[B31] Hair J. F. Black W. C. Babin B. J. Anderson R. E. (2010). Multivariate Data Analysis, 7th Edn. Upper Saddle River, NJ: Pearson.

[B32] Harman H. H. (1976). Modern Factor Analysis, 3rd Edn. Chicago, IL: University of Chicago Press.

[B33] He D. Guo Z. Zhang Y. (2022). The development of digital collection platform under responsible innovation framework. J. Open Innov. Technol. Mark. Complex. 8:203. doi: 10.3390/joitmc8040203

[B34] Henseler J. Ringle C. M. Sarstedt M. (2015). A new criterion for assessing discriminant validity in variance-based structural equation modeling. J. Acad. Mark. Sci. 43, 115–135. doi: 10.1007/s11747-014-0403-8

[B35] Horky F. Rachel C. Fidrmuc J. (2022). Price determinants of non-fungible tokens in the digital art market. Financ. Res. Lett. 48:103007. doi: 10.1016/j.frl.2022.103007

[B36] Hu L. Bentler P. M. (1999). Cutoff criteria for fit indexes in covariance structure analysis. Struct. Equ. Modeling 6, 1–55. doi: 10.1080/10705519909540118

[B37] Joselit D. (2021). NFTs, or the readymade reversed. October 176, 3–4. doi: 10.1162/octo_a_00419

[B38] Kapser S. Abdelrahman M. (2020). Acceptance of autonomous delivery vehicles for last-mile delivery in Germany. Transp. Res. Part C Emerg. Technol. 111, 210–225. doi: 10.1016/j.trc.2019.12.016

[B39] Kline R. B. (2023). Principles and Practice of Structural Equation Modeling, 5th Edn. New York, NY: Guilford Publications.

[B40] Li L. Zhan R. (2024). A study on decentralization and intellectual property rights construction strategies in NFT art: focusing on the Chinese case. Trans 16, 33–68. doi: 10.23086/trans.2024.16.02

[B41] Liang H. Saraf N. Hu Q. Xue Y. (2007). Assimilation of enterprise systems: the effect of institutional pressures and the mediating role of top management. MIS Q. 31, 59–87. doi: 10.2307/25148781

[B42] Limayem M. Hirt S. G. Cheung C. M. K. (2007). How habit limits the predictive power of intention: the case of information systems continuance. MIS Q. 31, 705–737. doi: 10.2307/25148817

[B43] Liu Y. Li H. Zhang W. Wang S. (2024). Understanding consumer adoption of short-video e-commerce: an extended UTAUT2 perspective. Electron. Commer. Res. Appl. 65:101385.

[B44] Lyubchenko I. (2022). NFTs and digital art: 21st century avant-garde impulse? *MC J*. 25. doi: 10.5204/mcj.2891. [Epub ahead of print].

[B45] Martinez B. M. McAndrews L. E. (2023). Do you take.? The effect of mobile payment solutions on use intention: an application of UTAUT2. J. Mark. Anal. 11, 458–469. doi: 10.1057/s41270-022-00175-6

[B46] Mekacher A. Bracci A. Nadini M. Martino M. Aiello L. M. Baronchelli A. (2022). Heterogeneous rarity patterns drive price dynamics in NFT collections. Sci. Rep. 12:13890. doi: 10.1038/s41598-022-17922-5 35974056 PMC9381539

[B47] Meng M. Li Z. Zhou T. Zhang W. (2024). Consumer usage of mobile visual search in China. J. Glob. Inf. Manag. 32, 1–29. doi: 10.4018/JGIM.349731

[B48] Migliore G. Wagner R. Cechella F. S. Liébana-Cabanillas F. (2022). Antecedents to the adoption of mobile payment in China and Italy: an integration of UTAUT2 and innovation resistance theory. Inf. Syst. Front. 24, 2099–2122. doi: 10.1007/s10796-021-10237-2 35095331 PMC8783184

[B49] Moorthy K. Yee T. T. Chun T'ing L. Vigneswari Kumaran V. (2019). Habit and hedonic motivation are the strongest influences in mobile learning behaviours among higher education students in Malaysia. Australas. J. Educ. Technol. 35, 174–191. doi: 10.14742/ajet.4432

[B50] Murray A. Kim D. Combs J. (2023). The promise of a decentralized internet: what is Web3 and how can firms prepare? Bus. Horiz. 66, 191–202. doi: 10.1016/j.bushor.2022.06.002

[B51] Mustafa S. Zhang W. Shehzad M. U. Anwar A. (2022). An integrated model of UTAUT2 to understand consumers 5G technology acceptance using SEM-ANN approach. Sci. Rep. 12:20056. doi: 10.1038/s41598-022-24532-8 36414788 PMC9681779

[B52] Muthe K. B. Sharma K. Sri K. E. N. (2020). “A blockchain based decentralized computing and NFT infrastructure for game networks,” in 2020 Second international conference on blockchain computing and applications (BCCA) (Antalya: IEEE), 73–77. doi: 10.1109/BCCA50787.2020.9274085

[B53] Nadini M. Alessandretti L. Di Giacinto F. Martino M. Aiello L. M. Baronchelli A. (2021). Mapping the NFT revolution: market trends, trade networks, and visual features. Sci. Rep. 11:20902. doi: 10.1038/s41598-021-00053-8 34686678 PMC8536724

[B54] Ong A. K. S. Prasetyo Y. T. Robles K. R. M. Persada S. F. Nadlifatin R. Chuenyindee T. . (2023). Determination of factors influencing the behavioral intention to play “Mobile Legends: Bang-Bang” during the COVID-19 pandemic. Sustainability 15:3170. doi: 10.3390/su15043170

[B55] Podsakoff P. M. MacKenzie S. B. Lee J.-Y. Podsakoff N. P. (2003). Common method biases in behavioral research: a critical review of the literature and recommended remedies. J. Appl. Psychol. 88, 879–903. doi: 10.1037/0021-9010.88.5.879 14516251

[B56] Podsakoff P. M. MacKenzie S. B. Podsakoff N. P. (2012). Sources of method bias in social science research and recommendations on how to control it. Annu. Rev. Psychol. 63, 539–569. doi: 10.1146/annurev-psych-120710-100452 21838546

[B57] Radermecker A.-S. V. Ginsburgh V. (2023). Questioning the NFT “revolution” within the art ecosystem. Arts 12:25. doi: 10.3390/arts12010025

[B58] Regner F. Urbach N. Schweizer A. (2019). “NFTs in practice – Non-fungible tokens as core component of a blockchain-based event ticketing application,” in ICIS 2019 Proceedings (Munich: Association for Information Systems (AIS)).

[B59] Schmitz A. Díaz-Martín A. M. Guillén M. J. Y. (2022). Modifying UTAUT2 for a cross-country comparison of telemedicine adoption. Comput. Hum. Behav. 130:107183. doi: 10.1016/j.chb.2022.107183 35017788 PMC8739826

[B60] Schomakers E.-M. Lidynia C. Ziefle M. (2022). Applying an extended UTAUT2 model to explain user acceptance of lifestyle and therapy mobile health apps. JMIR MHealth UHealth 10:e27095. doi: 10.2196/27095 35040801 PMC8808343

[B61] Shao X. Siponen M. (2011). Consumer acceptance and use of information technology: adding consumption theory to UTAUT2. Sprouts: Working Papers on Information Systems 11–157.

[B62] Sharma T. Zhou Z. Huang Y. Wang Y. Hui P. (2022). “It's a blessing and a curse”: unpacking creators' practices with NFTs and their communities. *arXiv* [preprint]. arXiv:2201.13233.

[B63] Shaw N. Sergueeva K. (2019). The non-monetary benefits of mobile commerce: extending UTAUT2 with perceived value. Int. J. Inf. Manag. 45, 44–55. doi: 10.1016/j.ijinfomgt.2018.10.024

[B64] Song C. Kim S. W. Sohn Y. (2020). Acceptance of public cloud storage services in South Korea: a multi-group analysis. Int. J. Inf. Manag. 51:102035. doi: 10.1016/j.ijinfomgt.2019.11.003

[B65] Suo W.-J. Goi C.-L. Goi M.-T. Sim A. K. S. (2022). Factors influencing behavioural intention to adopt the QR-code payment. Int. J. Asian Bus. Inf. Manag. 13, 1–22. doi: 10.4018/IJABIM.20220701.oa8

[B66] Talukder M. S. Sorwar G. Bao Y. Ahmed J. U. Palash M. A. S. (2020). Predicting antecedents of wearable healthcare technology acceptance by elderly: a combined SEM-neural network approach. Technol. Forecast. Soc. Change 150:119793. doi: 10.1016/j.techfore.2019.119793

[B67] Tamilmani K. Rana N. P. Wamba S. F. Dwivedi Y. K. (2021). The extended unified theory of acceptance and use of technology (UTAUT2): a systematic literature review and theory evaluation. Int. J. Inf. Manag. 57:102269. doi: 10.1016/j.ijinfomgt.2020.102269

[B68] Tan T. M. Salo J. Juntunen J. Kumar A. (2024). Consumer adoption of Web3 technologies: a systematic review and future research agenda. J. Bus. Res. 178:114674.

[B69] Technavio. (2022). Non-fungible Token (NFT) Market Size to Grow by USD 147.24. PR Newswire. Available online at: https://www.prnewswire.com/news-releases/non-fungible-token-nft-market-size-to-grow-by-usd-147-24—-technavio-301529125.html (Accessed July 15, 2025).

[B70] Tian Z. (2024). Reshaping the art market: blockchain technology, resale royalties, and the emerging Chinese paradigm. Arts Commun. 3:2875. doi: 10.36922/ac.2875

[B71] Tong A. (2022). Non-fungible token, market development, trading models, and impact in China. Asian Bus. Rev. 12, 7–16. doi: 10.18034/abr.v12i1.607

[B72] Truby J. Brown R. D. Dahdal A. Ibrahim I. A. (2022). Blockchain, climate damage, and death: policy interventions to reduce the carbon emissions, mortality, and net-zero implications of non-fungible tokens and Bitcoin. Energy Res. Soc. Sci. 88:102499. doi: 10.1016/j.erss.2022.102499

[B73] Uncovska M. Freitag B. Meister S. Fehring L. (2023). Patient acceptance of prescribed and fully reimbursed mHealth apps in Germany. J. Med. Syst. 47:14. doi: 10.1007/s10916-023-01910-x 36705853 PMC9880914

[B74] Valeonti F. Bikakis A. Terras M. Speed C. Hudson-Smith A. Chalkias K. (2021). Crypto collectibles, museum funding and OpenGLAM. Appl. Sci. 11:9931. doi: 10.3390/app11219931

[B75] Venkatesh V. Morris M. G. Davis G. B. Davis F. D. (2003). User acceptance of information technology: toward a unified view. MIS Q. 27, 425–478. doi: 10.2307/30036540

[B76] Venkatesh V. Thong J. Y. L. Xu X. (2012). Consumer acceptance and use of information technology: extending the unified theory of acceptance and use of technology. MIS Q. 36, 157–178. doi: 10.2307/41410412

[B77] Vishnu Prasad V. Girija S. Rakesh K. Prasanna Venkatesan S. (2024). What decides my purchase of non-fungible tokens? J. Consum. Behav. 23, 586–607. doi: 10.1002/cb.2221

[B78] Wang Q. (2023). Exploring the application value and path of NFT in the artwork industry. Adv. Econ. Manag. Polit. Sci. 17, 418–426. doi: 10.54254/2754-1169/17/20231133

[B79] Wang Y. Horky F. Baals L. J. Fidrmuc J. (2022). Bubbles all the way down? Detecting and date-stamping bubble behaviours in NFT and DeFi markets. J. Chin. Econ. Bus. Stud. 20, 415–436. doi: 10.1080/14765284.2022.2138161

[B80] Williams L. J. Hartman N. Cavazotte F. (2010). Method variance and marker variables: a review and comprehensive CFA marker technique. Organ. Res. Methods 13, 477–514. doi: 10.1177/1094428110366036

[B81] Xia Y. Wang C. Liu J. Zhang H. (2024). Digital art exhibitions and psychological well-being in Chinese generation Z. Humanit. Soc. Sci. Commun. 11:266. doi: 10.1057/s41599-024-02718-x

[B82] Xiao L. Guo Z. D'Ambra J. (2023). Understanding social commerce adoption in China: integrating UTAUT2 with social support theory. J. Retail. Consum. Serv. 72:103255.

[B83] Xu L. Lin J. Chan H. C. (2012). The moderating effects of utilitarian and hedonic values on information technology continuance. ACM Trans. Comput.-Hum. Interact. 19, 1–26. doi: 10.1145/2240156.2240160

[B84] Zarifis A. Castro L. A. (2022). The NFT purchasing process and the challenges to trust at each stage. Sustainability 14:16482. doi: 10.3390/su142416482

[B85] Zhang G. (2023). “Analysis of the current status of the NFT industry chain in China,” in Distributed, Ambient and Pervasive Interactions (Cham: Springer), 300–310. doi: 10.1007/978-3-031-34609-5_22

[B86] Zhang M. Liu Y. Wang S. Li H. (2024). Why do consumers engage in live streaming shopping? An extended UTAUT2 perspective in the Chinese context. J. Retail. Consum. Serv. 76:103584.

[B87] Zhao Z. (2021). Fulfilling the right to follow: using blockchain to enforce the artist's resale right. Cardozo Arts Entertain. Law J. 39, 239–274.

[B88] Zhou T. (2008). “The impact of perceived value on user acceptance of mobile commerce,” in 2008 International symposium on electronic commerce and security (Guangzhou: IEEE), 237–240. doi: 10.1109/ISECS.2008.9

